# Distinct functional relevance of dynamic GTPase cysteine methylation in fission yeast

**DOI:** 10.1038/s41598-017-06053-x

**Published:** 2017-07-20

**Authors:** Alejandro Franco, Teresa Soto, Rebeca Martín-García, Marisa Madrid, Beatriz Vázquez-Marín, Jero Vicente-Soler, Pedro M. Coll, Mariano Gacto, Pilar Pérez, José Cansado

**Affiliations:** 1 Yeast Physiology Group, Department of Genetics and Microbiology, Facultad de Biología, Universidad de Murcia, 30071 Murcia, Spain; 2 Instituto de Biología Funcional y Genómica (IBFG), Consejo Superior de Investigaciones Científicas/Departamento de Microbiología y Genética, Universidad de Salamanca, 37007 Salamanca, Spain

## Abstract

The final step in post-translational processing of Ras and Rho GTPases involves methylation of the prenylated cysteine residue by an isoprenylcysteine-O-carboxyl methyltransferase (ICMT). ICMT activity is essential for cell growth and development in higher eukaryotes, and inhibition of GTPase methylation has become an attractive target in cancer therapy to inactivate prenylated oncoproteins. However, the specificity and dynamics of the GTPase methylation process remain to be fully clarified. Notably, cells lacking Mam4, the ICMT ortholog in the fission yeast *Schizosaccharomyces pombe*, are viable. We have exploited this feature to analyze the role of methylation on GTPase localization and function. We show that methylation differentially affects GTPase membrane localization, being particularly relevant for plasma membrane tethering and downstream signaling of palmitoylated and farnesylated GTPases Ras1 and Rho2 lacking C-terminal polybasic motifs. Indeed, Ras1 and Rho2 cysteine methylation is required for proper regulation of differentiation elicited by MAPK Spk1 and for stress-dependent activation of the cell integrity pathway (CIP) and its main effector MAPK Pmk1. Further, Mam4 negatively regulates TORC2 signaling by a cross-inhibitory mechanism relying on Rho GTPase methylation. These results highlight the requirement for a tight control of GTPase methylation *in vivo* to allow adequate GTPase function.

## Introduction

Prenylation (i.e., modification by isoprenoid lipids) is a major post-translational modification that plays a critical role in regulating the localization and function of a number of proteins in eukaryotic organisms^1^. Ras and Rho-family GTPases are among the most prominent -CaaX protein members to become prenylated *in vivo*, and this change is essential for proper targeting to cellular membranes and biological activity^2, 3^. The first event in the prenylation process of Ras and Rho GTPases involves the covalent linkage of a farnesyl or geranylgeranyl isoprenoid lipid to a cysteine residue located at a conserved C-terminal tetrapeptide motif named the -CaaX box^4^. Once prenylated, the -aaX tripeptide is removed from the -CaaX box by proteolytic cleavage mediated by the endoplasmic reticulum RAS-converting -CaaX endopeptidase 1 (RCE1)^5^. Finally, the free carboxyl group of the isoprenylated cysteine is methylated by a specific ICMT, which is also located at the ER^5^. Other protein features, including the presence of a cluster of polybasic amino acids located upstream the -CaaX box, or additional modifications such as palmitoylation of cysteine residue/s, are often required to enhance and stabilize the Ras/Rho association to membranes^4^. *In vivo* protein palmitoylation by palmitoyltransferases (Protein Acyl Transferases, PATs) is a dynamic and reversible process that allows compartmentalization of GTPase membrane targeting and signaling^6^.

RCE1 and ICMT post-prenylation processing is essential for cell growth and development in higher eukaryotic organisms, and mice deficient in RCE1 or ICMT are embryonic lethal^7, 8^. Methylation is required for proper localization of Ras, but the involvement of ICMT function for membrane association of Rho GTPases has found different support^9, 10^. Pharmacological inhibition of ICMT leads to Ras mislocalization and EGF-induced stimulation of ERK MAPKs and Akt^11^, triggering disruption of the actin cytoskeleton and impaired activation of RhoA and Rac1^12^. Methylation also affects Rho proteins stability, although the effect is different depending on the GTPase^13, 14^. Remarkably, in contrast to prenylation and proteolysis, -CaaX protein methylation is a reversible process whose dynamic affect RhoA physiological function^15^. Moreover, the fact that both farnesylated and geranylgeranylated GTPases are exclusively methylated by ICMT *in vivo*, has converted this enzyme into a potential drug target to inhibit oncogenic Ras signaling^5^.

GTPases and the prenylation machinery are strongly conserved in lower eukaryotes like the budding yeast *Saccharomyces cerevisiae* ^16^ and the fission yeast *S*. *pombe* ^17^. In these organisms ICMT activity is encoded by STE14 and *mam4* ^+^ respectively^18^. Remarkably, either *ste14Δ* or *mam4Δ* mutants are viable and show no obvious defects other than the sterile phenotype^16, 18, 19^. Hence, contrary to mammalian cells, cysteine methylation might not be critical for the biological functions of -CaaX proteins in both organisms. Alternatively, a redundant methyltransferase might be involved in substrate methylation in the absence of canonical ICMT activity. In this work we demonstrate that Mam4 is the major ICMT activity present in fission yeast. We also show that impaired Mam4 function differentially affects Ras and Rho GTPase membrane localization, and that this leads to decreased activation of the sexual differentiation and cell integrity MAPK cascades, and enhanced TORC2-dependent downstream signaling. Therefore, cysteine methylation is biologically relevant for Ras and Rho GTPase signaling in this model organism.

## Results

### *mam4*^+^ encodes the major isoprenylcysteine-O-carboxyl methyltransferase activity responsible for Ras and Rho GTPase methylation ***in vivo***

**Figure 1:**
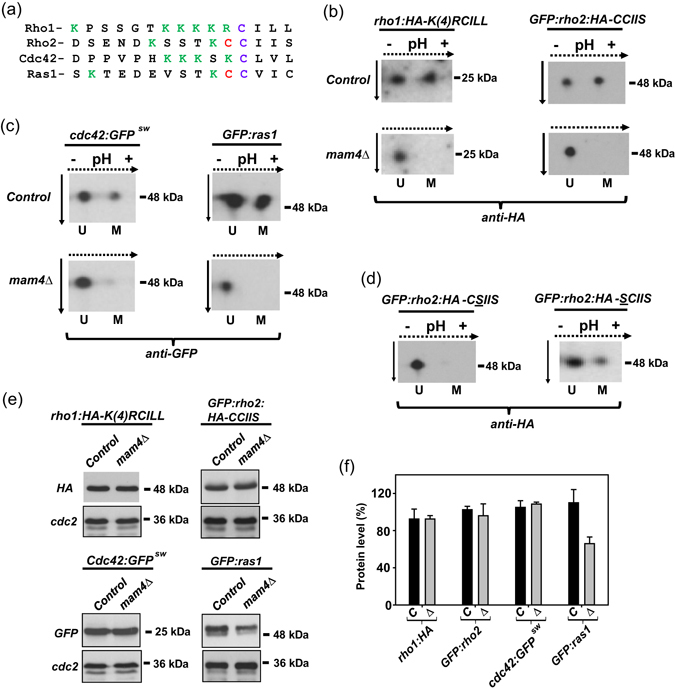
Mam4 I mediates cysteine methylation of Ras1 and Rho GTPases in fission yeast. (**a**) C-terminal sequences present in fission yeast GTPases Rho1, Rho2, Cdc42 and Ras1. Prenylated and palmitoylated cysteine residues are marked in blue and red, respectively. Positively charged residues are shown in green. (**b**) Cell extracts from control and *mam4∆* strains expressing Rho1-HA-K(4)RCILL or GFP-Rho2-HA-CCIIS fusions were subjected to isoelectric focusing and immunoblot analysis with anti-HA antibody. Results representative of two independent experiments are shown. U: unmethylated GTPase; M: methylated GTPase. Dotted arrows: direction of IEF; solid arrows: direction of SDS-PAGE separation. (**c**) Cell extracts from control and *mam4∆* strains expressing genomic Cdc42-GFP^SW^ or GFP-Ras1 genomic fusions were subjected to isoelectric focusing and immunoblot analysis with anti-GFP antibody. Results representative of two independent experiments are shown. U: unmethylated GTPase; M: methylated GTPase. Dotted arrows: direction of IEF; solid arrows: direction of SDS-PAGE separation. (**d**) Cell extracts from strains LSM502 (GFP-Rho2-HA-CSIIS*;* unprenylated GTPase) and LSM501 (GFP-Rho2-HA-SCIIS*;* unpalmitoylated GTPase), were analyzed as described in (**b**). (**e**) Cell extracts from growing cultures in YES medium of strains with the indicated genotypes were resolved by SDS-PAGE and hybridized separately with anti-GFP and anti-Cdc2 (loading control) antibodies. Results representative of two independent experiments are shown. (**f**) Quantification of protein levels (as mean ± SD) of GFP-GTPase fusions shown in (**e**). Black bars: control cells; gray bars: *mam4∆* cells.

A search in the fission yeast proteome (*http:*//*www*.*pombase*.*org*/) revealed the existence of 35 proteins showing *in vivo* prenylatable -CaaX, -CxC, or -CC motifs at their C-terminal ends (S1 Table). These include 17 GTPases of the Ras superfamily, including Rho GTPase members Rho1 to Rho5 and Cdc42; the Ras ortholog Ras1, mitochondrial GTPase Mss1, Rheb GTPase Rhb1, and Rab GTPases Ryh1, Ypt1 to Ypt5, Ypt7, and Ypt71 (S1 Table). Rho1, Cdc42, Rhb1, and Ypt1-3 are essential prenylated GTPases, whereas Rho2, Rho3, and Ras1 are both prenylated and palmitoylated *in vivo* (Fig. 1a)^20–23^. Rho1, Cdc42, and Ras1 are major regulators of morphogenesis, polarity, and sexual differentiation, while Rho2 and Rho1 are the two main upstream activators of the cell integrity MAPK pathway (CIP) in this organism^20, 24^. We employed isoelectric focusing coupled to Western blotting^15^, to detect both methylated and unmethylated GTPase isoforms in growing yeast cultures expressing either Rho1-HA-KKKKRCIIL, GFP-Rho2-HA-CCIIS, Cdc42-GFP^SW^, or GFP-Ras1 genomic fusions (Fig. 1b,c; S2 Table). It was previously demonstrated that the GFP-Rho2-HA fusion functionally complements the defective CIP signaling of a *rho2∆* mutant during vegetative growth^21^, whereas Cdc42-GFP^SW^ and GFP-Ras1 fusions are also fully functional *in vivo* ^25, 26^. We show here that C-terminal HA-tagging did not compromise Rho1 function, as seen by its ability to suppress thermosensitivity and growth sensitivity to Caspofungin of the hypomorphic Rho1 allele *rho1-596* ^27^ (Suppl. Figure S1). A mixture of methylated and unmethylated species (~1:1 ratio) was present in control cells expressing the GTPase fusions described above (Fig. 1b,c), but only the unmethylated isoform was detected in extracts from mutants lacking the ICMT ortholog Mam4 (*mam4∆* cells). Rho2 is farnesylated and palmitoylated *in vivo* within its C-terminal motif at the cysteine-197 and -196, respectively (Fig. 1a)^21, 28^. Replacement of cysteine-197 by serine fully blocked Rho2 methylation (GFP-Rho2-HA-CSIIS; Fig. 1d), which agrees with the dogma that this modification requires prior prenylation^4^. On the contrary, GTPase methylation was still evident in cells expressing a prenylated and non-palmitoylated Rho2 (GFP-Rho2-HA-SCIIS; Fig. 1d). Notably, while protein levels of Rho1-HA, GFP-Rho2-HA and Cdc42-GFP^SW^ fusions were virtually identical in control and *mam4∆* cells (Fig. 1e,f), GFP-Ras1 levels were reduced in the *mam4∆* mutant cells (~60% of the control; Fig. 1e,f). Altogether, these results strongly suggest that Ras1 and Rho GTPases naturally occur as a mixture of methylated and -unmethylated isoforms, and that *mam4* ^+^ encodes the major isoprenylcysteine-O-carboxyl methyltransferase activity responsible for their *in vivo* methylation. They also suggest that cysteine methylation positively regulates Ras1 stability.

### Mam4 function differentially affects Ras and Rho GTPase localization at the plasma membrane

**Figure 2:**
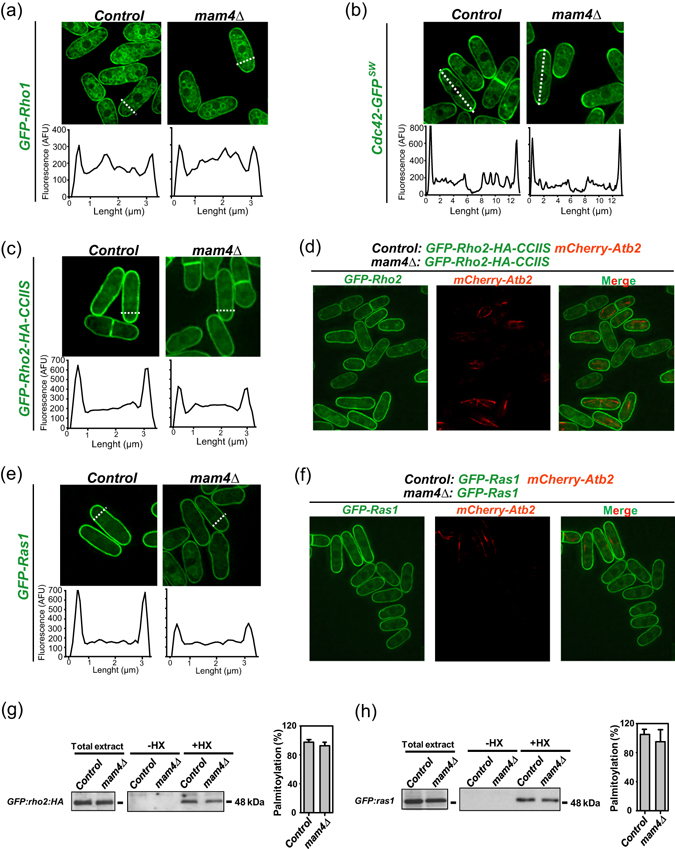
Mam4 function differentially affects GTPase localization at the plasma membrane. (**a**) Deconvolved images of cells from control and *mam4∆* strains expressing GFP-Rho1 fusions grown in YES medium and observed by fluorescence microscopy. Representative fluorescence intensity plots (as arbitrary fluorescence units) were generated from line scans across the cell width (dotted white lines). (**b**) Images of cells from control and *mam4∆* strains expressing Cdc42-GFP^SW^ genomic fusions observed by fluorescence microscopy. Representative fluorescence intensity plots were generated from line scans across the cell length (dotted white lines). (**c**) Images of cells from control and *mam4∆* strains expressing GFP-Rho2-HA-CCIIS genomic fusions observed by fluorescence microscopy. Representative fluorescence intensity plots were obtained as described in (**a**). (**d**) Deconvolved images of mixed control (GFP-Rho2-HA-CCIIS, mCherry-Atb2) and *mam4∆* (GFP-Rho2-HA-CCIIS) cells were observed by fluorescence microscopy. (**e**) Images of cells from control and *mam4∆* strains expressing GFP-Ras1 genomic fusions observed by fluorescence microscopy. Representative fluorescence intensity plots were obtained as described in (**a**). (**f**) Deconvolved images of mixed control (GFP-Ras1, mCherry-Atb2) and *mam4∆* (GFP-Ras1) cells were observed by fluorescence microscopy. (**g**) Rho2 palmitoylation assayed by the acyl-biotinyl switch assay in cell lysates from control and *mam4∆* strains expressing a GFP-Rho2-HA genomic fusion. Biotinylation is specific for proteins containing a free sulfhydryl generated after hydroxylamine cleavage (+HX). Total extracts from the strains were included as loading controls. GFP-Rho2-HA fusion was detected employing anti-HA antibody. Percentage of palmitoylation (as mean ± SD) in control and *mam4∆* cells was determined from biological duplicates. (**h**) Ras1 palmitoylation by the acyl-biotinyl switch assay in control and *mam4∆* strains expressing a GFP-Ras1 genomic fusion was determined as above. GFP-Ras1 fusion was detected employing anti-GFP antibody. Percentage of palmitoylation (as mean ± SD) in control and *mam4∆* cells was determined from biological duplicates.

Once shown that ICMT activity is executed by Mam4 in fission yeast, we explored the relevance of Mam4 function on GTPase membrane targeting by comparative analyses of the subcellular localization of the GFP-tagged Cdc42, Rho2, and Ras1 versions described above in control versus *mam4Δ* cells. Since the GFP-Rho1 fusion was not fully functional and failed to completely suppress thermosensitivity of a *rho1-596* background (Suppl. Figure S1), this construct was expressed in a strain carrying the endogenous *rho1* ^+^ gene. The previously reported localization at plasma membrane, cell tips, and/or endomembranes of GFP-fused Rho1 and Cdc42^29, 30^ was not significantly affected in cells lacking Mam4 (Fig. 2a,b). Indeed, by analyzing the fluorescence from line scans across the width (GFP-Rho1) and length (Cdc42-GFP) of cells in the early-mid G2 phase of the cell cycle, we confirmed that the ratio of cortical versus internal GFP fluorescence of both GTPases remained unaffected in *mam4∆* cells (Fig. 2a,b). Contrariwise, the plasma membrane targeting of Rho2 and Ras1^21, 23^ was significantly reduced in cells lacking Mam4 as compared to control cells (Fig. 2e–e) (ratio of cortical/internal GFP-Rho2: 1.214 ± 0.05 for wild type cells versus 0.8490 ± 0.15 for *mam4∆* cells; GFP-Ras1: 1.411 ± 0.10 for wild type cells versus 0.684 ± 0.09 for *mam4∆* cells). Microscopic observation of mixed control and *mam4∆* cells expressing each of the above GFP-fused constructs, and control cells also expressing mCherry-fused alpha tubulin (mCherry-Atb2; internal control for discrimination between both strains), further confirmed the positive impact of Mam4-dependent methylation on plasma membrane targeting of both Rho2 and Ras1 (Fig. 2d–f).

Both Rho2 and Ras1, but not Rho1 and Cdc42, are palmitoylated *in vivo*, and this lipid modification is essential for proper plasma membrane localization^21, 23^. However, the Rho2 and Ras1 palmitoylation levels, as determined by a modified version of the acyl-biotinyl switch assay, were similar in control and *mam4Δ* cells (Fig. 2g,h), supporting that impaired palmitoylation is not the reason for their decreased plasma membrane localization in *mam4Δ* cells.

### Mam4 mediates proper plasma membrane tethering of palmitoylated and farnesylated GTPases lacking polybasic motifs

**Figure 3:**
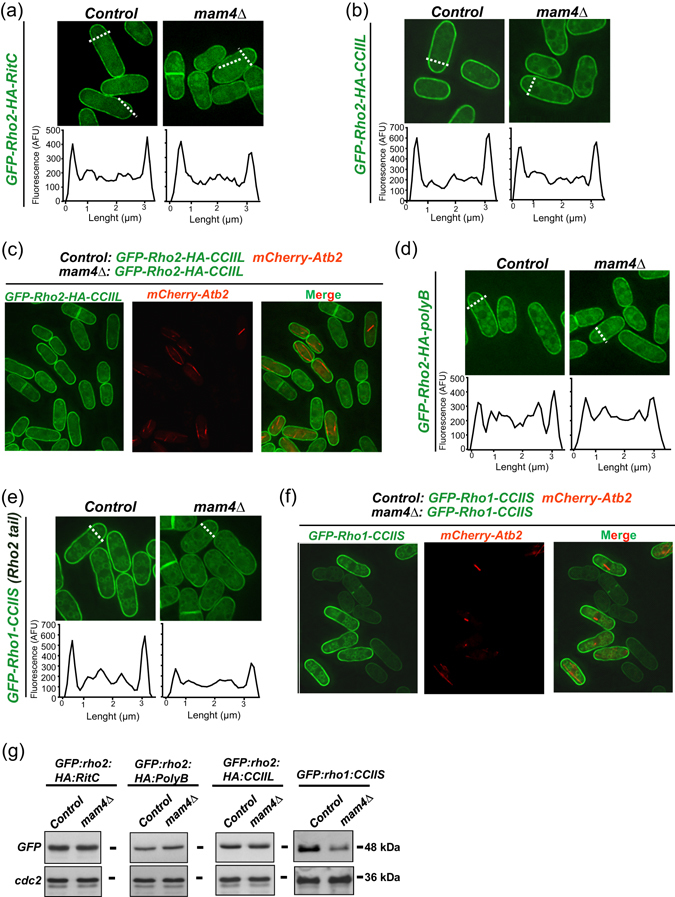
Mam4 mediates proper plasma membrane tethering of palmitoylated and farnesylated GTPases lacking polybasic motifs. (**a**) Deconvolved images of cells from control and *mam4∆* strains expressing genomic unprenylated GFP-Rho2-HA-RitC fusions grown in YES medium and observed by fluorescence microscopy. Representative fluorescence intensity plots (as arbitrary fluorescence units) were generated from line scans across the cell width (dotted white lines). (**b**) Images of cells from control and *mam4∆* strains expressing genomic geranylgeranylated and palmitoylated GFP-Rho2-HA-CCIIL fusions observed by fluorescence microscopy. Representative fluorescence intensity plots were obtained as described in (**a**). (**c**) Deconvolved images of mixed control (GFP-Rho2-HA-CCIIL, mCherry-Atb2) and *mam4∆* (GFP-Rho2-HA-CCIIL) cells were observed by fluorescence microscopy. (**d**) Images of cells from control and *mam4∆* strains expressing genomic polybasic, farnesylated and palmitoylated GFP-Rho2-HA-*polyB* fusions observed by fluorescence microscopy. Representative fluorescence intensity plots were obtained as described in (**a**). (**e**) Images of cells from control and *mam4∆* strains expressing genomic farnesylated and palmitoylated GFP-Rho1-CCIIS fusions (Rho2 tail) were observed by fluorescence microscopy. Representative fluorescence intensity plots were obtained as described in (**a**). (**f**) Deconvolved images of mixed control (GFP-Rho1-CCIIS, mCherry-Atb2) and *mam4∆* (GFP-Rho1-CCIIS) cells were observed by fluorescence microscopy. (**G**) Cell extracts from growing cultures in YES medium of strains with the indicated genotypes were resolved by SDS-PAGE and hybridized separately with anti-GFP and anti-Cdc2 (loading control) antibodies. Results representative of two independent experiments are shown.

Replacement of the wild type Rho2 C-terminal -CaaX motif by the last 25 amino acids from the hydrophobic C-terminus of the mammalian plasma membrane non-lipidated GTPase RitC^21^ bypassed the need of Mam4 for proper targeting to the plasma membrane (Fig. 3a). Hence, specific structural features within -CaaX motifs may mediate Mam4-dependent plasma membrane localization of Rho2 and Ras1. Indeed, both GTPases are farnesylated *in vivo* ^21, 23^, whereas Rho1 and Cdc42 are geranylgeranylated^31^. Additionally, Cdc42 and Rho1 harbor C-terminal polybasic sequences before the -CaaX box which are missing in Rho2 and Ras1 (Fig. 1a). We thus tested the significance of these motifs for plasma membrane targeting in absence of methylation. Replacement of the Rho2 terminal serine residue within the -CaaX box by leucine (Rho2-*CCII* *L*) bypasses the farnesylation requirement of the wild type GTPase, which becomes geranylgeranylated and fully targeted to the plasma membrane, with no evident signaling defects^21, 28^. As shown in Fig. 3b, plasma membrane tethering of geranylgeranylated Rho2 was only slightly reduced in *mam4Δ* cells as compared to control cells. This was accompanied by a small decrease in the ratio of cortical versus internal GFP fluorescence (1.070 ± 0.05 for wild type cells versus 0.942 ± 0.04 for *mam4∆* cells), which clearly was less pronounced than in cells expressing the wild type and farnesylated Rho2 version (Fig. 2c). Observation of mixed control and *mam4Δ* cultures confirmed that the difference in membrane targeting of geranylgeranylated Rho2 was indeed very small (Fig. 3c). Inclusion of the Rho1 polybasic sequence within the Rho2 C-terminal motif (*KKKKR* *CCIIS;* Rho2-polyB chimera) eliminated that small difference and resulted in a similar membrane targeting in control and *mam4Δ* cells (Fig. 3d). Remarkably, replacement of the C-terminal motif of Rho1 GTPase by that of Rho2 (GFP-Rho1-*KSSTKCCIIS* chimera) decreased its plasma membrane targeting (Fig. 3e,f), and the ratio of cortical versus internal GFP fluorescence in a *mam4Δ* background (0.930 ± 0.08 for wild type cells versus 0.6252 ± 0.03 for *mam4∆* cells). Moreover, total protein levels of this chimera were reduced in the *mam4∆* mutant by ~60% as compared to those in control cells, but remained unchanged in the other constructs (Fig. 3g). As a whole, these findings indicate that methylation is particularly relevant for proper plasma membrane tethering and stabilization of palmitoylated and farnesylated GTPases lacking C-terminal polybasic amino acids.

### Mam4 regulates MAPK signaling elicited by palmitoylated, plasma membrane tethered Ras1

**Figure 4:**
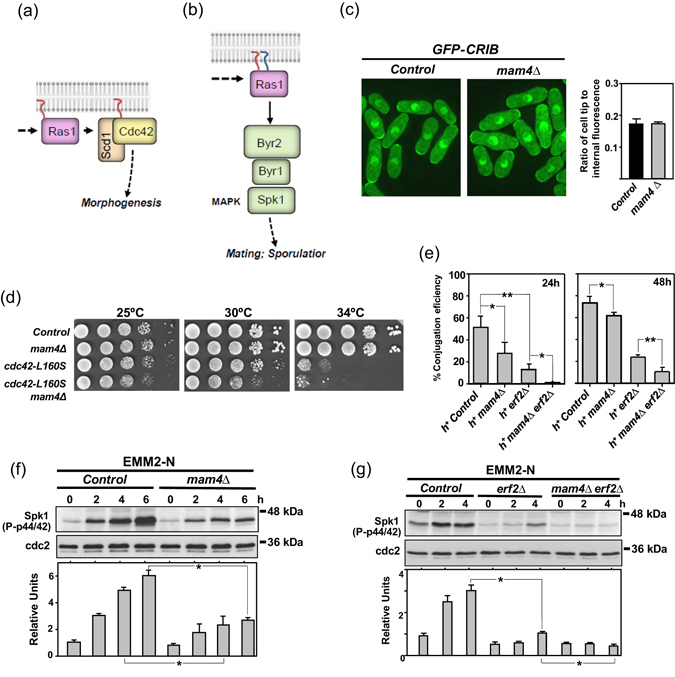
Cysteine methylation is important to regulate sexual differentiation mediated by palmitoylated Ras1. (**a**) In fission yeast cellular morphogenesis is regulated by unpalmitoylated Ras1 from endomembranes. (**b**) Pheromone signaling is modulated by plasma membrane-tethered and palmitoylated Ras1 to activate MAPK Spk1. (**c**) Deconvolved images of cells from control and *mam4∆* strains expressing a GFP-CRIB fusion (GTP-bound Cdc42) were grown in YES medium and observed by fluorescence microscopy. (**d**) Serial dilutions of suspensions of control, *mam4∆*, Cdc42*-L160S*, and *mam4∆* Cdc42*-L160S* strains were spotted on YES plates and incubated at either, 25, 30, and 34 °C for 3 days. Results representative of three independent experiments are shown. (**e**) Control, *mam4∆*, *erf2∆*, and *mam4∆ erf2∆* strains of the h^+^ mating type were mixed with wild type h^−^ cells, poured on SPA plates, and incubated at 25 °C. The percentage of conjugation efficiency (as mean ± SD) was determined after 24 and 48 h of incubation by microscopic counting of number of vegetative cells, zygotes, and asci. Biological triplicate samples (≥300 cells) were counted for each cross. (**f**) Control and *mam4∆* strains were grown in EMM2 medium and transferred to the same medium lacking nitrogen source. TCA extracts were obtained from samples taken at different times, and activated Spk1 was detected with anti-phospho-p44/42 antibody. Relative units as mean ± SD for Spk1 activation (biological triplicates) were determined with respect to the internal control (anti-Cdc2 blot) at each time point.*P < 0.05. (**G**) Spk1 activation in cultures from control, *erf2∆*, and *mam4∆ erf2∆* strains was detected and quantified as described in (**f**). *P < 0.05.

Although Mam4 may encode the only ICMT activity present in fission yeast, *mam4Δ* cells are viable^18^, suggesting that methylation of prenylated cysteines is not essential. To analyze the biological relevance of methylation in this organism, we compared several known signaling outputs which are dependent on the activity of Ras and Rho GTPases in control and *mam4Δ* cells. Ras signaling is spatially segregated in eukaryotic organisms^32^. Indeed, in fission yeast, cellular morphogenesis is regulated by unpalmitoylated Ras1 localized to the endomembranes, whereas mating pheromone signaling is dependent on palmitoylated Ras1 located to the plasma membrane (Fig. 4a,b)^25^. Endomembrane targeted Ras1 interacts with Scd1, a GDP-GTP exchange factor for Cdc42 to maintain cell polarity^33^. We observed that both cortical and internal localization of active GTP-bound Cdc42 (GFP-CRIB)^34^ was identical in control and *mam4Δ* cells (Fig. 4c). Moreover, the thermosensitive phenotype of a strain expressing the hypomorphic Cdc42 allele Cdc42-*L160S* ^35^ was not intensified by simultaneous deletion of Mam4 (Fig. 4d). These results indicate that Mam4 function does not have a significant impact on the Cdc42-dependent morphogenetic regulation by unpalmitoylated Ras1.

Palmitoylated plasma membrane Ras1 controls sexual differentiation by a mechanism that includes binding and activation of Byr2, a MAPKKK for the pheromone signaling pathway whose main element is MAPK Spk1 (Fig. 4b)^36^. It was initially described that *mam4Δ* cells of the h^+^ mating type do not show conjugation defects^18^. However, a close monitoring of this process revealed that, as compared to h^+^ control cells, h^+^ *mam4Δ* cells were partially defective during mating when crossed with wild type h^−^ cells (Fig. 4e). We found that an anti-phospho P44/42 antibody routinely employed to detect phosphorylation of the cell integrity MAPK Pmk1^37^, was also suitable to detect Spk1 phosphorylation during nitrogen starvation in extracts prepared under denaturing conditions (Suppl. Figure S2). It has been shown that the Byr2-Byr1-Spk1 MAPK module becomes activated in response to both nitrogen starvation and pheromone signals^38^. Indeed, we observed that the low levels of dually phosphorylated Spk1 shown by h^+^ cells growing in minimal EMM2 medium increased progressively when shifted to the same medium but lacking the nitrogen source (EMM2-N; Fig. 4f). Importantly, the increase in Spk1 phosphorylation was also considerably less pronounced in nitrogen-starved *mam4Δ* cells (Fig. 4f). These results, together with the observation that plasma membrane targeting of palmitoylated Ras1 is reduced in *mam4Δ* cells (Fig. 2a,b), are consistent with a model where Mam4 modulates nitrogen and mating pheromone signaling by palmitoylated Ras1. The class III DHHC palmitoyl transferase Erf2, ortholog to human zDHHC9, palmitoylates fission yeast Ras1 *in vivo* ^23^. However, an Erf2-independent mechanism may also be involved in Ras palmitoylation, since this GTPase is partially palmitoylated and is targeted to the membrane in *erf2Δ* cells^23^. Spk1 activation in response to nitrogen starvation was impaired in *erf2Δ* cells as compared to control cells (Fig. 4g). This activation was further reduced in *erf2Δ mam4Δ* cells (Fig. 4g), and resulted in a stronger decrease in conjugation efficiency in comparison to *erf2Δ* cells (Fig. 4e). Therefore, cysteine methylation is important for the regulation of sexual differentiation mediated by Erf2-dependent and -independent palmitoylated Ras1.

### Mam4 function is required for proper downstream activation of the cell integrity MAPK pathway elicited by Rho1 and Rho2, and for cross-inhibition of TORC2 signaling

**Figure 5:**
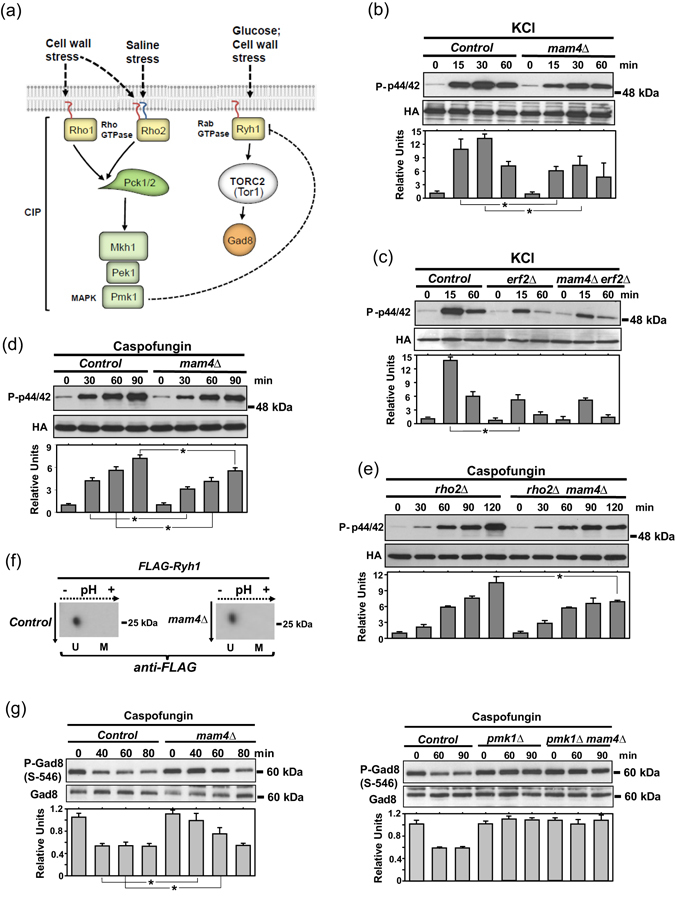
Mam4 positively regulates signaling and activation of the cell integrity pathway (CIP) elicited by Rho1 and Rho2, and cross-inhibition of TORC2 signaling. (**a**) Activation of the main CIP effector, MAPK Pmk1, in response to saline stress is totally dependent on the activity of palmitoylated Rho2, whereas MAPK activation in response to cell wall stress is channeled through Rho1 and Rho2. In turn, activated Pmk1 negatively regulates Ryh1-TORC2-Gad8 signaling. (**b**) Growing cultures of control and *mam4Δ* strains expressing genomic Pmk1-HA6H fusions were treated with 0.6 M KCl for the indicated times. Pmk1-HA6H fusion was purified by affinity chromatography, and activated/total Pmk1 detected with anti-phospho-p44/42 and anti-HA antibodies, respectively. Relative units as mean ± SD for Pmk1 activation (biological triplicates) were determined with respect to the internal control (anti-HA blot) at each time point. *P < 0.05. (**c**) Growing cultures of control *erf2∆*, and *mam4∆ erf2∆* strains expressing genomic Pmk1-HA6H fusions were treated with 0.6 M KCl, and activated/total Pmk1 was detected and quantified as described in (**b**). *P < 0.05. (**d**) Growing cultures of control and *mam4Δ* strains expressing genomic Pmk1-HA6H fusions were treated with 1 μg/ml Caspofungin, and activated/total Pmk1 was detected and quantified as described in (**b**). *P < 0.05. (**e**) Growing cultures of *rho2Δ* and *rho2∆ mam4∆* strains expressing genomic Pmk1-HA6H fusions were treated with 1 μg/ml Caspofungin, and activated/total Pmk1 was detected and quantified as described in (**b**). *P < 0.05. (**f**) Cell extracts from control and *mam4∆* strains expressing a FLAG-Ryh1 fusion were subjected to isoelectric focusing and immunoblot analysis with anti-FLAG antibody. U: unmethylated GTPase; M: methylated GTPase. Dotted arrows: direction of IEF; solid arrows: direction of SDS-PAGE separation. Results representative of two independent experiments are shown. (**g**) Control and *mam4∆* strains (left panels), and control, *pmk1∆*, and *pmk1∆ mam4∆* strains (right panels) were grown in YES medium and treated with 1 μg/ml Caspofungin. Cell extracts were resolved by SDS-PAGE and S546-phosphorylated and total Gad8 detected with anti-phospho-S546 and anti-Gad8 antibodies, respectively. Relative units as mean ± SD for Gad8 phosphorylation (biological triplicates) were determined with respect to the internal control (anti-Gad8 blot) at each time point. *P < 0.05.

In fission yeast activation of cell integrity pathway (CIP) MAPK Pmk1 induced by osmotic saline stress is totally dependent upon the signaling mediated by Rho2 GTPase (Fig. 5a)^39^. As compared to control cells, deletion of *mam4*+ induced a moderate but reproducible decrease in the magnitude of Pmk1 phosphorylation during stress induced with KCl (Fig. 5b). Thus, cysteine methylation is functionally relevant for Rho2 signaling to the CIP in response to this stimulus. Similar to Ras1, both Erf2-dependent and -independent mechanisms are involved in Rho2 palmitoylation *in vivo* ^21^. However, *erf2Δ* cells displayed a low Pmk1 activation in response to saline stress with no further decrease in a double *erf2Δ mam4Δ* mutant (Fig. 5c). Therefore, cysteine methylation is relevant for downstream signaling to the CIP elicited only by Erf2-palmitoylated Rho2.

The CIP MAPK also becomes activated in response to cell wall damage induced by the β-glucan synthase inhibitor Caspofungin, and such activation signal is transduced to the MAPK module via both Rho1 and Rho2 GTPases^24^. Progressive Pmk1 activation in control cells in the presence of Caspofungin was decreased in *mam4Δ* cells, likely due to a flawed Rho2 signal (Fig. 5d). Notably, defective Pmk1 activation was aggravated in a double *rho2Δ mam4Δ* mutant as compared to *rho2Δ* cells at long incubation times (Fig. 5e). These results suggest that ICMT activity may also regulate Rho1 functions in fission yeast.

Fission yeast has two TOR complexes, TORC1 and TORC2. The small Rab GTPase Ryh1, ortholog to human Rab6, is the main TORC2 activator, which includes the nonessential catalytic subunit Tor1 (Fig. 5a)^40^. Ryh1 has a prenylatable -CxC motif (S1 Table) suggesting that its activity towards TORC2 might be influenced by Mam4 function. However, only the unmethylated isoform was present in extracts from both control and *mam4∆* cells expressing a genomic and fully functional FLAG-Ryh1 fusion^40^, supporting that this GTPase is not methylated *in vivo* (Fig. 5f). The AGC-kinase Gad8 (Akt ortholog) is the major target for TORC2, and becomes phosphorylated at S546 within the hydrophobic motif by Tor1 in the presence of glucose (Fig. 5g)^41^. This phosphorylation becomes partially reduced after 30 minutes of glucose starvation or in response to a cell wall stress with Caspofungin (Fig. 5g)^41, 42^. Notably, Gad8-S546 phosphorylation only decreased moderately in *mam4∆* cells after 40 to 80 minutes in Caspofungin-treated cells as compared to control cells (Fig. 5g). Pmk1 activity negatively impacts Ryh1-TORC2 signaling, and Gad8-S546 phosphorylation levels remain elevated in *pmk1∆* cells during prolonged incubation in response to a cell wall stress or in absence of glucose^42^ (Fig. 5g). Considering that Pmk1 activation by Rho1 and Rho2 during stress is reduced in *mam4∆* cells, these results suggest that Mam4 might cross-inhibit TORC2 signaling by regulating the methylation status of Rho1 and/or Rho2. In agreement with this idea, Gad8-S546 phosphorylation in *pmk1∆* cells treated with Caspofungin was not further enhanced by simultaneous deletion of Mam4 (Fig. 5g).

## Discussion

The final step in post-translational processing of prenylated proteins, including Ras and Rho GTPases, involves methylation of the prenylated cysteine by an ICMT methyltransferase^5^. In mammalian cells ICMT is the only enzyme that catalyses the carboxylmethylation of prenylated proteins^1^, and early work in fission yeast demonstrated that mutants lacking the single ICMT ortholog Mam4 showed no detectable methyltransferase and produced farnesylated but totally unmethylated M factor^18^. By employing a isoelectric focusing (IEF)-immunoblot approach, in this work we demonstrate that members of the Ras GTPase superfamily including Rho1, Rho2, Cdc42, and Ras1 become methylated *in vivo* by Mam4, and that methylated GTPase isoforms are fully absent in *mam4∆* cells. Although the existence of a redundant Mam4-independent ICMT activity cannot be totally discarded due to limitations in the IEF assay and the low number of prenylated proteins assayed, previous and current evidences strongly suggest that Mam4 encodes the major ICMT present in fission yeast, and that a single protein of this class is present along the eukaryotic lineage.

Earlier studies performed with mouse embryonic fibroblasts (MEFs) lacking ICMT proposed that this activity is required for proper localization of farnesylated Ras but not geranylgeranylated Rho proteins^9^. However, later work showed that loss of ICMT had significant impact on the subcellular localization and membrane association of geranylgeranylated Rho GTPases^10^. Here we found that ICMT activity is particularly relevant for the tethering to the plasma membrane of Rho2 and Ras1, which are farnesylated and palmitoylated *in vivo* ^21, 23^. The different effect of cysteine methylation on membrane localization might rely on the prenylation mode of both GTPases, since their palmitoylation level was not altered in *mam4Δ* cells. However, our results reveal a more complex scenario where both farnesylation and the absence of a polybasic motif near the -CaaX box entail a need for ICMT (Mam4) activity to promote efficient GTPase plasma membrane tethering. This conclusion is based upon three main observations. First, plasma membrane tethering of a geranylgeranylated Rho2 chimera was, at most, slightly reduced in *mam4Δ* cells as compared to the wild type and farnesylated GTPase. Second, replacement of the Rho1 -CaaX box for that of Rho2 strongly reduced plasma membrane targeting of the chimeric GTPase in a *mam4Δ* background. Third, inclusion of the Rho1 polybasic motif upstream of the Rho2 -CaaX box bypassed the need for Mam4 to promote its efficient plasma membrane localization. In contrast to Ras1 or Rho2, the presence of a cluster of polybasic amino acids located upstream the -CaaX box in Rho1 or Cdc42 might compensate for the negative charge resulting from the lack of cysteine methylation in absence of ICMT activity, thus favoring electrostatic interaction with acidic membrane lipids.

C-terminal methylation differentially affects stability of mammalian Ras and Rho GTPases. Methylation increases the half-life of RhoA and decreases that of RhoB, whereas inactivation of ICMT retards the turnover and increases the steady-state levels of total Ras proteins^13^. In contrast, in this work we found that Ras1 protein levels are reduced in *mam4Δ* cells, suggesting that its stability is positively regulated by Mam4. Intriguingly, while protein levels of Rho2 remained unaltered in *mam4Δ* cells, the levels of a Rho1 chimera fused to the Rho2 -CaaX box were reduced in absence of Mam4. The farnesylated and palmitoylated C-terminal sequences of both Rho2 and Ras1 are strongly conserved (Fig. 1a). Therefore, the presence of additional GTPase-specific structural elements might be responsible for the differential effect of methylation on the steady-state protein levels of both GTPases.

Methylation of -CaaX proteins is a reversible process, and negative control of methylation by carboxylesterase family member CES1 affects RhoA’s physiological function^15^. Indeed, CES1 silencing or ICMT overexpression increased RhoA activity and prompted strong changes in cytoskeletal organization in breast cancer cells^15^. In this study we found that not only Rho1 (RhoA ortholog) and Cdc42, but also Rho2 and Ras1 are present in fission yeast as pools of methylated and unmethylated isoforms at a ~1:1 ratio. This suggests that regulatory and dynamic methylation is a common theme among GTPases and may not be restricted to specific family members. Overexpression of the wild type *mam4* ^+^ gene caused a clear growth defect and cells displayed an altered morphology with increased width and engrossed septa (Suppl. Figure S3). However, all of these phenotypes were replicated in cells overexpressing the inactive allele Mam4-*H168A R205A*, in which two critical and conserved amino acid residues involved in ICMT cofactor (H168) and prenyl lipid substrate binding (R205)^43^ were replaced by alanine (Suppl. Figure S3). Therefore, the deleterious effect of increased Mam4 expression in fission yeast does not result from increased ICMT activity, but is an indirect consequence due to protein toxicity.

While ICMT activity is essential in mammalian cells for cell growth and development^7^, *mam4Δ* mutants are viable and do not show apparent defects in polarized cell growth and morphology^18^ (this work). Thus, at first sight, this postprenylation mechanism might be considered an evolutionary relic that plays a dispensable role in fission yeast GTPase function. However, the absence of a redundant ICMT activity provided an opportunity to precisely address the impact, if any, of cysteine methylation on GTPase biological functions in an evolutionary ancient eukaryotic model. Indeed, as discussed below, our results reveal a scenario where prenylcysteine methylation in this organism impacts GTPase function in subtle but clear ways. This impact was evidenced by the downstream signaling defects displayed by unmethylated Ras1 and Rho2 GTPases, and reveals the requirement for a control of the GTPase methylation threshold to modulate their function *in vivo*.

Plasma membrane localization of Ras2, one of the two Ras paralogs present in budding yeast, is decreased in *ste14Δ* (ICMT-less) cells, but this mutant did not exhibit detectable impairment of Ras function or cell viability^16^. However, in this work we show that fission yeast ICMT function is important for activation of the pheromone signaling MAPK pathway that is regulated by plasma membrane bound Ras1^44^. This was evidenced by a marked decrease in the phosphorylation threshold of MAP kinase Spk1 during nitrogen withdrawal and in the conjugation efficiency in *mam4Δ* versus control cells. Importantly, the positive role of cysteine methylation on Ras1 function is limited to the palmitoylated plasma membrane-bound GTPase, and it was not observed in unpalmitoylated Ras1 that localizes to endomembranes and regulates morphogenesis via Cdc42^25^. Ras signaling is also spatially segregated in higher eukaryotes, where unpalmitoylated pools of H- and N-Ras isoforms have been shown to signal from endomembranes, including Golgi apparatus or endoplasmic reticulum^45^. Hence, our findings reveal that cysteine methylation may exert a differential impact on Ras function depending on its specific membrane localization.

Cysteine methylation is also important for the function of the Rho GTPases RhoA and Rac1 in mammalian cells^12^. We observed here that cysteine methylation has a differential effect on Rho GTPases localization and/or function. Similar to Ras1, ICMT activity has a relevant role in eliciting adequate plasma membrane localization of Rho2 and activation of cell integrity MAP kinase Pmk1 in response to stress. Although we could not observe significant changes in membrane localization of Rho1, methylation may also play a role in Rho1 function, since Mam4 deletion further decreased the low Pmk1 activation of *rho2Δ* cells in response to cell wall stress, which is transmitted to the MAPK module via Rho1^24^. In addition, both the thermosensitive and Caspofungin sensitive phenotypes of a strain expressing the hypomorphic Rho1 allele *rho1-596* ^27^ were exacerbated in a *rho1-596 mam4Δ* double mutant background (Suppl. Figure S4). Likely, changes in Rho1 localization pattern in control versus *mam4Δ* cells are too subtle to be detected with the GFP-Rho1 allele that is not fully functional.

Fission yeast Erf2 palmitoyl transferase is mainly responsible for the *in vivo* palmitoylation of both Ras1 and Rho2, although an Erf2-independent mechanism is also involved in this process^21, 23^. We observed that cysteine methylation was important for the regulation of sexual differentiation by both Erf2-dependent and -independent palmitoylated Ras1. On the contrary, Mam4 deletion only reduced the activation of Pmk1 in response to stress prompted by Erf2-palmitoylated Rho2. Again, these results highlight the different effects of ICMT activity on GTPase function.

In mammalian cells ICMT processing is required for Rheb GTPase localization, but is dispensable for Rheb-induced activation of S6 kinase through mTOR^46^. We found that in response to glucose availability, changes in Rhb1-TORC1 dependent phosphorylation of Psk1 and Rps6, the respective S6 kinase and ribosomal protein S6 orthologs in fission yeast^47^, were identical in control and *mam4Δ* cells (Suppl. Figure S5). Therefore, as in mammalian cells, lack of ICMT activity does not influence Rheb-TOR signaling in fission yeast. Since ICMT also methylates the -CxC class of mammalian isoprenylated Rab proteins^7^, the increase in TORC2 signaling observed in *mam4∆* cells under glucose starvation could be initially interpreted as a negative influence of methylation of the Rab GTPase member Ryh1 in TORC2 signaling. To this date, Ryh1 is the only known example of a TORC2 activator within this class of proteins^48^. Instead, our observations confirmed that Ryh1 is not methylated *in vivo*, and support that increased TORC2 signaling in cells lacking Mam4 might result from a cross-inhibitory mechanism due to impaired methylation of Rho1 and/or Rho2, and the ensuing drop in Pmk1 activity.

The GTPase methylation step is an attractive target in cancer therapy, since both farnesylated and geranylgeranylated proteins are modified *in vivo* by ICMT, so that its inhibition might downregulate the function of prenylated oncoproteins^1^. The transforming ability of oncogenic K-Ras and activated Raf kinase has been eliminated by conditional ICMT deletion, and inhibitors of ICMT enzymatic activity have shown promising activity in both a variety of cancer cell lines *in vitro* and human cancer xenograft models *in vivo* ^1^. In this context, we found that *mam4Δ* cells are growth sensitive to Camptothecin, a potent DNA topoisomerase I inhibitor (Suppl. Figure S4)^49^. Several Camptothecin analogues are currently used in cancer therapy^49^, raising the possibility that combined use of both ICMT and Camptothecin derivatives might improve their anticancer efficacy during chemotherapy. The existence of powerful genetic and genomic tools could make fission yeast a complementary and potentially useful model organism to identify new players involved in the dynamic control of cysteine methylation, to search for additional cellular targets whose inhibition is lethal in absence of ICMT function (i.e., through synthetic lethal screens), and to study the biological impact of methylation on GTPase function.

## Methods

### Strains, growth conditions, and gene disruption

The *S*. *pombe* strains used in this work are listed in Supplementary S2 Table. They were grown with shaking at 28 °C in rich (YES) or minimal (EMM2) medium with 2% glucose, and supplemented with adenine, leucine, histidine, or uracil (100 mg/L, Sigma Chemical). Mutant strains were obtained either by standard transformation procedures or by mating followed by random spore analysis. The *mam4* ^+^ null mutants were obtained by entire deletion of the corresponding coding sequences and their replacement with the G418 (*kanR*) or nourseothricin (*natR*) cassettes by PCR-mediated strategy using plasmids pFA6a-*kanMX6* or pFA6a-*natMX6* as templates, respectively^50^. Strains expressing the *mam4*+ gene under the control of the strong (3X) thiamine inducible promoter (*nmt1*)^51^ were grown in liquid EMM2 with thiamine (5 mg/L), and transferred to the same medium lacking thiamine for 24-48 hours. In osmotic-saline and cell-wall stress experiments log-phase cultures (OD_600_ = 0.5) were supplemented with either KCl (Sigma-Aldrich) or Caspofungin (Sigma-Aldrich), respectively. In nitrogen starvation experiments cells grown in EMM2 medium were recovered by filtration and resuspended in medium lacking nitrogen source (ammonium chloride). In glucose starvation experiments cells grown in YES medium with 7% glucose were recovered by filtration, and resuspended in the same medium lacking glucose and osmotically equilibrated with 3% glycerol.

### Quantification of mating efficiency

Equivalent amounts (~10^8^ cells) of strains of the opposing mating type were mixed, poured on SPA plates, and incubated at 25 °C. The mating efficiency was determined after 24 and 48 h of incubation by microscopic counting of number of vegetative cells (V), zygotes (Z), and asci (A), according to the following equation:

$$ \% \,{\rm{mating}}\,{\rm{efficiency}}=(2{\rm{Z}}+2{\rm{A}})\times 100/(2{\rm{Z}}+2{\rm{A}})+{\rm{V}}.$$

Triplicate samples (at least 300 cells each) were counted for each cross.

### Gene fusion and site-directed mutagenesis

To construct integrative plasmid pIL-rho1:HA:K(4)RCILL, the *rho1* ^+^ ORF plus regulatory sequence was amplified by PCR using fission yeast genomic DNA as template and employing the 5′-oligonucleotide PRho1-5 (ACTTA*GCGGCCGC*TTCTATATTCCTGCTATG, which hybridizes at positions 508 to 490 upstream of the *rho1* ^+^ ATG start codon and contains a *Not*I site), and the 3′-oligonucleotide Rho1HA:RCILL-3 (ACTTA*CCCGGG*TTACAACAAGATACAACGCTTCTTCTTCTTAGTGCCCGCATAGTCAGGAACATCGTATGGGTAGCCTCCACTAGAGGGCTTCACTTTGG), which hybridizes at the 3′ end of *rho1* ^+^ ORF and incorporates a 63 nucleotide sequence (underlined) encoding one HA epitope (sequence GYPYDVPDYAG), followed by the ten C-terminal aminoacids of Rho1 GTPase (sequence TKKKKRCILL), and a *Sma*I site. The resulting PCR fragment was digested with *Not*I and *Sma*I and cloned into the integrative plasmid pIL-GFP^37^. Integrative plasmids expressing GFP-fused Rho2:PolyB (C-terminal aminoacids sequence K*KKK*KCCII) was obtained by PCR using plasmid pIL-GFP-rho2:HA:CCIIS as template^21^, the 5′ oligonucleotide PRho2-5 (CCTTA*TCTAGA*TCACGGGTCTGCGTTGGC; contains a *Xba*I site), and the mutagenic 3′ oligonucleotide Rho2:PolyB-3 (ACTTA*CCCGGG*TTATGAAATGATGCAGCATTT**CT**T**TT**T**CT**TCTTGCCCGCATAGTC; contains a *Sma*I site; base changes are indicated in bold). To construct integrative plasmids expressing wild-type or chimeric N-terminal green fluorescent protein (GFP)-fused versions of Ras1 and Rho1 under the control of its natural promoters, we followed a modular PCR based approach. PCR fragments including 5′ regulatory plus ORF sequences were amplified using fission yeast genomic DNA as the template in a first-round reactions using the following oligonucleotide pairs: PRas1-5 (CCTTA*TCTAGA*GAAACTACATCCTTAACG; contains a *Xba*I site) and Ras1GFP2-3 (TTCTCCTTTACTCATCACTATTTTATAAAGC; the underlined sequence is complementary to 5′ end in GFP ORF) to amplify Ras1 5′regulatory sequence; Ras1GFP3-5 (GAACTATACAAACATATGAGGGTAAGTCTA; the underlined sequence is complementary to 3′ end in GFP ORF) and Ras1-3 (ACTTA*GGATCC*ATGCTGGTATGTCGTTTCTTG; contains a *BamH*I site), to amplify Ras1 ORF; PRho1-5 and Rho1GFP2-3 (TTCTCCTTTACTCATCCCTAGATTTGTTTACT), to amplify Rho1 5′ regulatory sequence; Rho1GFP3-5 (GAACTATACAAACATATGGCGACAGAACTTC) and Rho1-3 (ACTTA*GGATCC*GTTGAATGTGCTTCGACTG; contains a *BamH*I site), to amplify Rho1 ORF. The above fragments were gel purified and used in a second-round PCR in the presence of plasmid pGFT41 (GFP donor) as template and the correspondent external oligonucleotides. The resulting PCR fragments were digested with either *Xba*I and *BamH*I (Ras1), or *Not*I and *BamH*I (Rho1), and cloned into pIL-GFP to obtain plasmids pIL-GFP-Ras1 and pIL-GFP-Rho1. To construct integrative plasmid expressing N-terminal GFP fused version of Rho1 with Rho2 tail (pIL-GFP-rho1:CCIIS; C-terminal aminoacids sequence KSSTKCCIIS), the PCR reaction included plasmid pIL-GFP-Rho1 as template, the 5′-oligonucleotide PRho1-5, and the 3′oligonucleotide Rho1CCIIS-3 (ACTTA*CCCGGG*TTATGAAATGATGCAGCATTTTGTAGAACTCTTTCCACTAGAGGGCTTCACTTTGG; contains a *Sma*I site). The purified PCR fragment was digested with *Not*I and *Sma*I and cloned into plasmid pIL-GFP. The above integrative plasmids were digested at the unique *Nru*I site within *leu1*+, and the linear fragments were transformed into MI200, *ras1Δ* or *rho2Δ* strains. *leu1*+ transformants were obtained, and the correct integration of the fusions was verified by both PCR and Western blot analysis. Wild-type Mam4 overexpression constructs were obtained by PCR amplification of the corresponding ORF using yeast genomic DNA as template with the 5′oligonucleotide Mam4-5 (TATAT*CTCGAG*ATGGGGAATTTACATA; contains a *Xho*I site) and the 3′oligonucleotides Mam4-3 (TATAT*GGATCC*CTATGGAATTAAGGGA; contains a *BamH*I site). A Mam4 inactive allele (Mam4-*H168A R205A*) was obtained by sequential PCR site-directed mutagenesis using wild type Mam4 as template and the 5′oligonucleotides Mam4-H168A-5 (GCTTACGTTAGA**GC**CCCATCATACGTT; base changes are indicated in bold), and Mam4-R205A-5 (TTTTTCTCACAG**GC**AATTACTACCGAA), and the 3′oligonucleotides Mam4-H168A-3 (AACGTATGATGG G**GC**TCTAACGTAAGC) and Mam4-R205A-3 (TTCGGTAGTAATT**GC**CTGTGAGAAAAA). The purified PCR products were digested with *Xho*I and *BamH*I and cloned into the expression plasmids pREP3X and pREP4X^45^.

### Detection of methylated/unmethylated GTPases by isoelectric focusing

Isoelectric focusing of yeast extracts was adapted from a previously described method^15^. Briefly, yeast cell cultures cells were lysed in buffer A (10% glycerol, 50 mM Tris HCl pH 7.5, 150 mM NaCl, 0.1% Nonidet NP-40, plus specific proteases inhibitor, Sigma Chemical). The lysate was cleared by centrifugation for 10 min at 13,000 rpm at 4 °C, and protein concentration was determined. Equal amounts of protein, generally 1 mg, were precipitated. Protein pellets were resuspended in rehydration buffer (8 M urea, 1% Chaps, 50 mM DTT, 0.2% biolytes (Bio-Rad, Bio-Lyte 3/10), 0.001% bromophenol blue), and samples were loaded onto Bio-Rad ReadyStrip IPG strips (11 cm, pH 5–8) for separation. Following isoelectric focusing, proteins were resolved in SDS-PAGE gels and transferred to Hybond-ECL membranes. Rho1-HA and GFP-Rho2-HA fusions were detected by immunoblot analysis with a mouse monoclonal anti-HA antibody (12CA5, Roche Molecular Biochemicals). Cdc42-GFP^SW^ and GFP-Ras1 fusions were detected with mouse monoclonal anti-GFP antibody (Roche). FLAG-Ryh1 fusion was detected with mouse monoclonal anti-FLAG antibody (Sigma-Aldrich). Immunoreactive bands were revealed with anti-mouse-HRP-conjugated secondary antibodies (Sigma-Aldrich) and the ECL system (GE-Healthcare).

### Detection of total and activated Pmk1

Cell extracts were prepared under native conditions employing chilled acid-washed glass beads and lysis buffer (10% glycerol, 50 mM Tris HCl pH 7.5, 15 mM Imidazole, 150 mM NaCl, 0.1% Nonidet NP-40, plus specific protease and phosphatase inhibitor, Sigma Chemical). Affinity chromatography purification of HA-tagged Pmk1 with Ni^2+^-NTA-agarose beads (Qiagen), and SDS-PAGE was performed as described^52^. Dual phosphorylation in Pmk1 was detected employing rabbit polyclonal anti-phospho-p44/42 (Cell Signaling). Total Pmk1 was detected with mouse monoclonal anti-HA antibody. Immunoreactive bands were revealed with anti-rabbit or anti-mouse-HRP-conjugated secondary antibodies (Sigma-Aldrich) and the ECL system (GE-Healthcare).

### Detection of total and activated Spk1

Cells from yeast cultures were fixed and total protein extracts were prepared by precipitation with trichloroacetic acid (TCA) as previously described^53^. Proteins were resolved in 10% SDS-PAGE gels and transferred to Hybond-ECL membranes. Dual phosphorylation in Spk1 was detected employing rabbit polyclonal anti-phospho-p44/42 (Cell Signaling). Mouse monoclonal anti-PSTAIR (anti-Cdc2, Sigma-Aldrich) was used for loading control. Immunoreactive bands were revealed with anti-rabbit or anti-mouse HRP-conjugated secondary antibodies (Sigma), and the ECL system (GE-Healthcare).

### Detection of total and phosphorylated Psk1, Gad8 and Rps6

Total and phosphorylated Psk1 levels were detected in strains expressing Psk1-13myc fusions with a monoclonal mouse anti-c-myc antibody (clone 9E10, Roche Molecular Biochemicals). S546 phosphorylated and total Gad8 were detected with specific anti-phospho-S546 and anti-Gad8 rabbit polyclonal antibodies (GenScript). Immunoreactive bands were revealed with anti-rabbit HRP-conjugated secondary antibody (Sigma) and the ECL system (GE-Healthcare). Phosphorylated Rps6 was detected by employing phospho-(Ser/Thr) Akt substrate (PAS) antibody (Cellular Signaling). Immunoreactive bands were revealed with anti-rabbit or anti-mouse HRP-conjugated secondary antibodies (Sigma) and the ECL system (GE-Healthcare).

### Detection of *in vivo* palmitoylation with the acyl-biotinyl switch assay

*S*. *pombe* strains expressing Rho2:HA and GFP:Ras1 alleles were grown in YES (100 ml) to a final OD_600_ = 0.8. Cell from 50 ml of cultures were resuspended in 1 ml lysis buffer (50 mM HEPES pH 7.4, 150 mM NaCl, 5 mM EDTA, 0.2% Triton X-100) containing 10 mM N-ethylmaleimide (NEM; Sigma Chemical) plus protease inhibitors (Sigma Chemical). Cell extracts were processed exactly as described^54^, including NEM removal by repeated chloroform-methanol precipitation, treatment with or without 0.7 M Hydroxylamine in the presence of HPDP-Biotin (Thermo Scientific), and recovery of acyl-biotinylated with Streptavidin Agarose beads (Thermo Scientific). After washings, the proteins were eluted in Laemmli sample buffer, subjected to SDS-PAGE, and analysed by Western blot with either mouse monoclonal anti-HA antibody, or mouse monoclonal anti-GFP antibody (Roche) as described above.

### Quantification of *Western* blot experiments and reproducibility of results

Densitometric quantification of Western blot signals as of 16-bit.jpg digital images of blots was performed using ImageJ^55^. Relative Units for Pmk1 activation were estimated by determining the signal ratio of the anti-phospho-P44/42 blot (activated Pmk1) with respect to the anti-HA blot (total Pmk1) at each time point. Relative Units for Spk1 activation were estimated by determining the signal ratio of either anti-phospho-P44/42 blot (activated Spk1) with respect to the anti-cdc2 blot (internal control) at each time point. Unless otherwise stated, results shown correspond to experiments performed as biological triplicates. Mean relative units ± SD and/or representative results are shown. *P*-values were analyzed by unpaired Student’s *t* test.

### Plate assay of stress sensitivity for growth

*S*. *pombe* control and mutant strains were grown in YES liquid medium to OD_600_ = 0.5. Appropriate decimal dilutions were spotted per duplicate on YES solid medium or in the same medium supplemented with different concentrations of Caspofungin (Sigma) or Camptothecin (Sigma). Plates were incubated at 25, 30, or 34 °C for 3 days and then photographed. All the assays were repeated at least three times with similar results. Representative experiments are shown in the corresponding Figures.

### Fluorescence microscopy

Images of GFP-fused GTPases were obtained with an Olympus 1X71 microscope equipped with a personal Delta Vision System and a Photometrics CoolSnap HQ2 camera. Stacks of 5 z-planes, 0.2 um apart, were acquired across the cells width. Images were then deconvolved using the Softworx software from Applied Precission. All fluorescence images shown correspond to a single- middle plane- from these z-series after deconvolution. Fluorescence distribution of cortical (plasma membrane or cell tips) versus internal GFP intensity through cells (n ≥ 15 cells) was determined with ImageJ by producing line scans across the cell width or length with the *plot profile* tool. Once the background signal from image was subtracted, the ratio of cortical to cytoplasmic fluorescence signal was calculated by averaging the plot values corresponding to the two cortical peaks and dividing by the average of data values within the inner cell. Calcofluor white was employed for cell wall/septum staining.

## Electronic supplementary material

Supplementary information
