# Supplementary material for: Distinct functional relevance of dynamic GTPase cysteine methylation in fission yeast
Source: Sci Rep. 2017 Jul 20;7:6057. doi: 10.1038/s41598-017-06053-x (PMC5519673; doi:10.1038/s41598-017-06053-x)
Supplement: Supplementary file 1 — Supplementary information [file 41598_2017_6053_MOESM1_ESM.pdf]

Supplemental material for:

Distinct functional relevance of dynamic GTPase cysteine methylation in fission yeast.

Alejandro Franco, Teresa Soto, Rebeca Martín-García, Marisa Madrid, Beatriz Vázquez-Marín, Jero Vicente-Soler, Pedro M. Coll, Mariano Gacto, Pilar Pérez, and José Cansado.

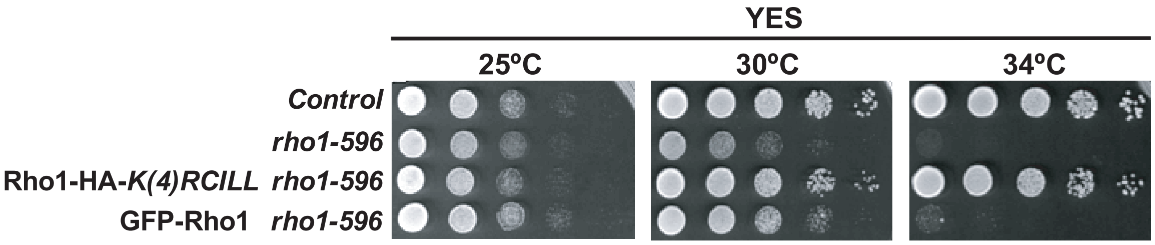

Supplementary Figure S1: **Functional characterization of cells expressing Rho1-HA-*K(4)RCILL* and GFP-Rho1 fusions.**

Serial dilutions of suspensions of strains MI200 (control), LS201 (*rho1-596*), AFS013 (Rho1-HA-*K(4)RCILL rho1-596*), and AFS025 (GFP-Rho1 *rho1-596*) were spotted on YES plates and incubated for 3 days at either 25, 30, and 34ºC. Results representative of three independent experiments are shown.

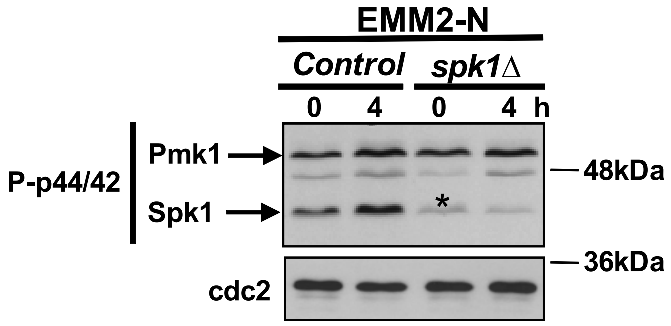

Supplementary Figure S2: Detection of Spk1 phosphorylation *in vivo*.

Control and *spk1*Δ strains were grown in EMM2 medium and transferred to the same medium lacking nitrogen source. TCA extracts were obtained from samples taken at the times indicated, and phosphorylated Pmk1 and Spk1 (arrows) were detected with anti-phospho-p44/42 antibody. Note the existence of a minor non-specific band (asterisk) which migrates with a relative size almost identical to that of phosphorylated Spk1 in extracts from *spk1*Δ cells, and whose intensity slightly decreases relative to the internal loading control (anti-Cdc2) along the course of the experiment. Results from a representative experiment are shown.

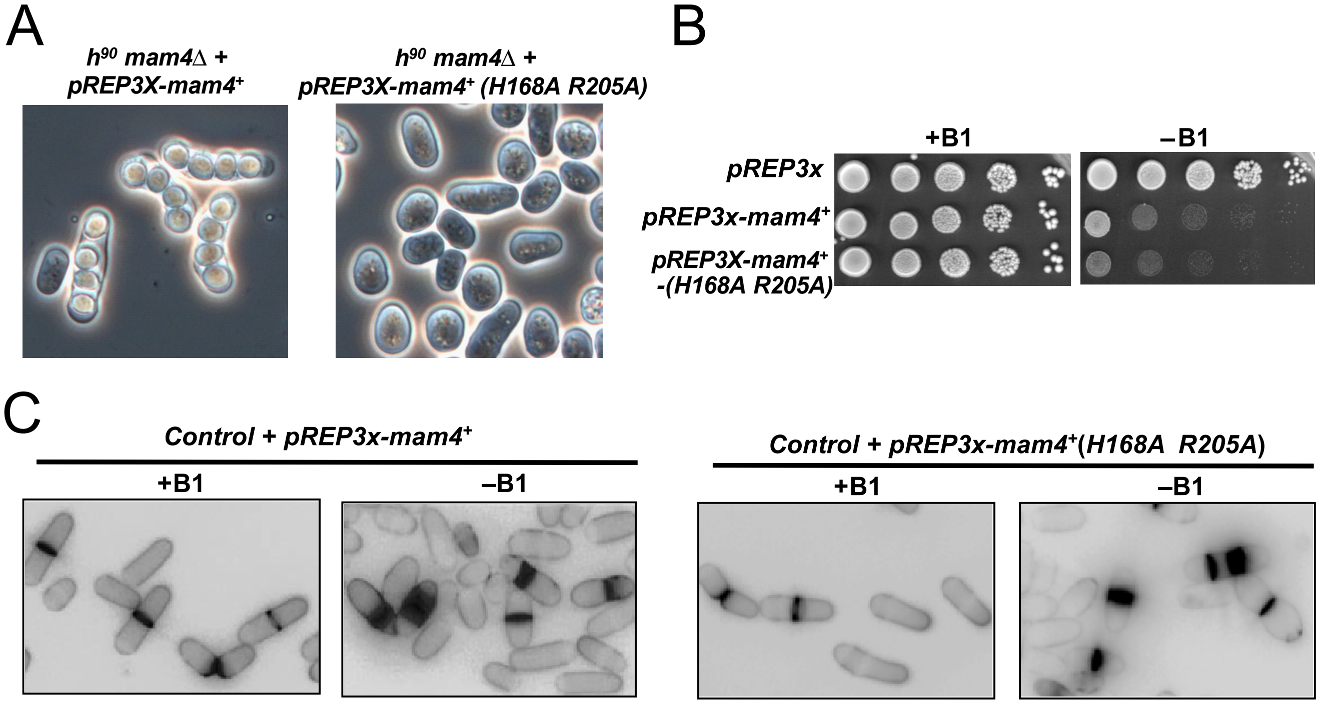

Supplementary Figure S3: Deleterious and toxic effects associated to Mam4 overexpression are unrelated to increased ICMT activity.

(**A**) A h^{90} *mam4*Δ strain was transformed separately with plasmids pREP3X-*mam4*$^{+}$ (wild type Mam4), and pREP3X-*mam4*$^{+}$(*H168A R205A*) (functionally inactive Mam4). The respective transformants were spotted on SPA plates with 0.1 µg/ml thiamine (B1), incubated for 36 hours at 25ºC, and observed by phase contrast microscopy.

(**B**) Control strain was transformed separately with pREP3X, pREP3X-*mam4*$^{+}$, and pREP3X-*mam4*$^{+}$ (*H168A R205A*) plasmids, and serially diluted suspensions of the respective transformants were spotted on EMM2 plates with or without 5 µg/ml thiamine, and incubated for 5 days at 30ºC. Results representative of three independent experiments are shown.

(**C**) Cultures from control strain separately transformed with pREP3X-*mam4*$^{+}$ and pREP3X-*mam4*$^{+}$ (*H168A R205A*) plasmids were grown in EMM2 medium with or without thiamine for 24 hours, and the cells were observed by fluorescence microscopy after staining with calcofluor white.

A

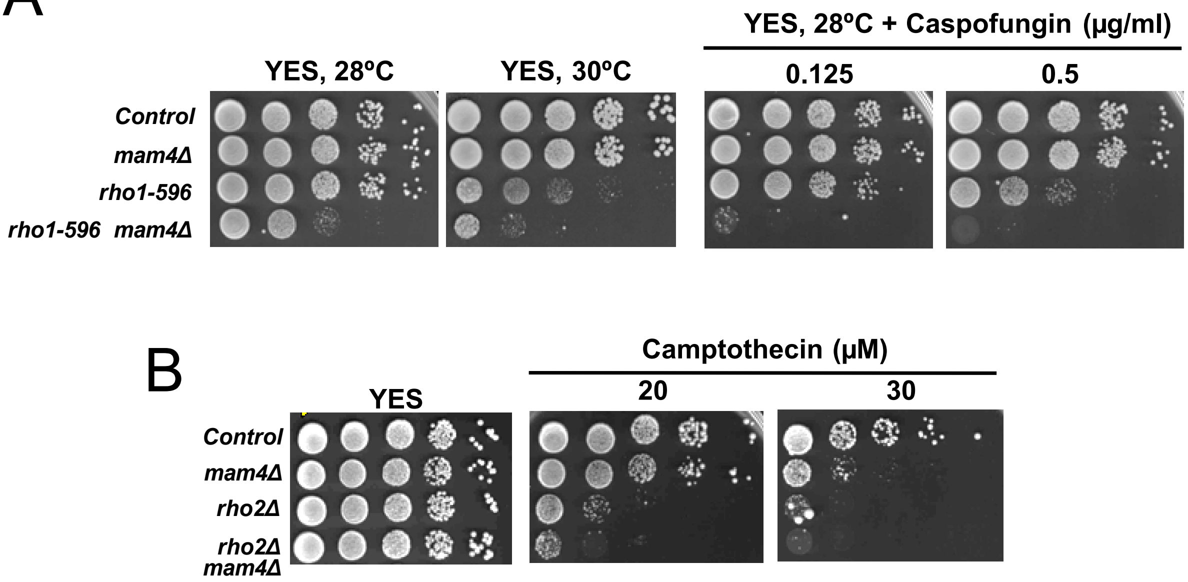

Supplementary Figure S4

(A) Mam4 deletion aggravates the phenotypes of the hypomorphic Rho1 mutant *rho1-596*.

Serial dilutions of suspensions of control, *mam4*Δ, *rho1-596*, and *mam4*Δ *rho1-596* strains were spotted on YES plates and incubated for 3 days at either 28 or 30ºC, and at 28ºC with different concentrations of Caspofungin. Results representative of three independent experiments are shown.

(B) *mam*Δ cells are sensitive to Camptothecin.

Serial dilutions of suspensions of control, *mam4*Δ, *rho2*Δ*,* and *rho2*Δ *mam4*Δ strains were spotted on YES plates supplemented with different concentrations of Camptothecin and incubated at 30ºC for 3 days. Results representative of three independent experiments are shown.

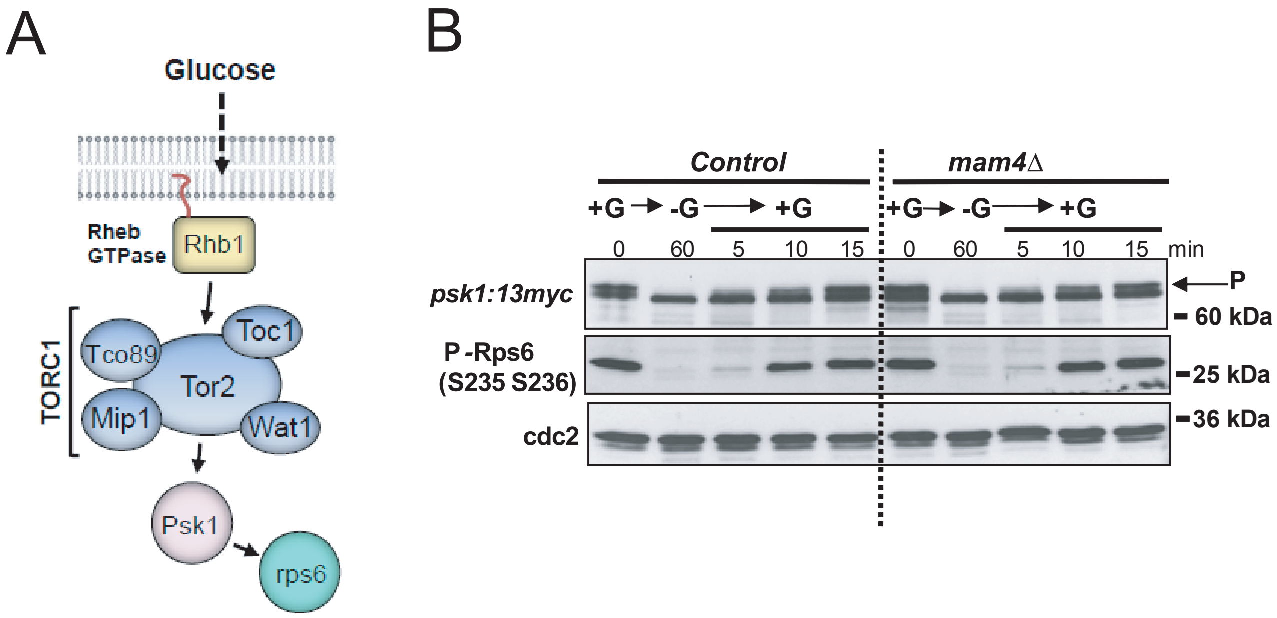

Supplementary Figure S5: **Mam4 function does not impact Rhb1-TORC2 signaling.**

(**A**) In fission yeast Rheb GTPase ortholog Rhb1 regulates TORC1 activity in response to glucose availability to phosphorylate the S6 kinase ortholog Psk1, which in turn phosphorylates the ribosomal protein S6 ortholog Rps6.

(**B**) Control and *mam4Δ* strains were grown in YES medium at 25ºC, starved for glucose during 60 minutes (-G), and then resuspended in YES medium with glucose for the indicated times. The Psk1-13myc fusion was detected after incubation with anti-myc antibodies. P: TORC1-phosphorylated Psk1. Rps6 phosphorylation was detected with phospho-(Ser/Thr) Akt substrate (PAS) antibody. Anti-cdc2 immunobloting was used as a loading control.

Supplementary Table S1: Proteins in the fission yeast proteome harbouring C-terminal CaaX/CXC/CC motifs [a].

Systematic ID	Product	C-terminal CaaX/CXC/CC motif
SPAC1F7.04	Rho family GTPase Rho1	-SGTKKKKRCILL
SPAC16.01	Rho family GTPase Rho2	-NDKSSTKCCIIS
SPAC23C4.08	Rho family GTPase Rho3	-ADESHGTGCIIA
SPAC16A10.04	Rho family GTPase Rho4	-SFSFSKKSCVIL
SPAC20H4.11c	Rho family GTPase Rho5	-PKTKKKKHCILL
SPAC110.03	Rho family GTPase Cdc42	-VPHKKKSKCLVL
SPAC17H9.09c	GTPase Ras1	-EDEVSTKCCVIC
SPAC222.05c	Mitochondrial GTPase Mss1	-FSVIFSKFCVGK
SPBC428.16c	Rheb GTPase Rhb1	-SPPGDGKGCVIA
SPAPB8E5.05	M-factor precursor Mfm1	-YTPKVPYMCVIA
SPAC513.03	M-factor precursor Mfm2	-YTPKVPYMCVIA
SPBPJ4664.03	M-factor precursor Mfm3	-YTPKVPYMCVIA
SPAC24B11.10c	Chitin synthase regulatory factor-like Cfh1 (predicted)	-PKKKQQEQCVVM
SPCC417.05c	Chitin synthase regulatory factor Cfh2	-KRSKNRESCIIS
SPBC1289.01c	1,3-beta-glucan synthase regulatory factor Chf3/Chr4	-KFLIKHNKCIIS
SPBC530.04	Tea1 anchoring protein Mod5	-GSKLEKFCCILM
SPAC30D11.11	Haemolysin-III family protein (predicted)	-ETDYEAFSCGVL
SPAC6B12.07c	Ubiquitin-protein ligase E3 (predicted)	-SVVSGQNNCVIM
SPAC23H3.09c	Threonine aldolase Gly1 (predicted)	-IVAKPGEFCVGY
SPAC12G12.11c	DUF544 family protein	-KSRKQSENCLIS
SPAC3C7.05c	alpha-1,6- mannanase (predicted)	-SHGKKDKDCVIS
SPBC725.10	Mitochondrial transport protein, tspO homolog (predicted)	-AGYLNLGYCLLN
SPAC11H11.03c	ATP-dependent polydeoxyribonucleotide 5'-hydroxyl-kinase activity implicated in DNA repair (predicted)	-CFPRLKTCCAIM
SPAC607.09c	Battenin CLN3 family protein	-QADRGRDWCALT
SPBC13G1.11	SNARE Ykt6 (predicted)	-SAKKQNSCCIIA
SPAC4C5.02c	Rab GTPase Ryh1	-IQPNENESSCNC
SPAPB1A10.10c	Rab GTPase Ypt71	-SKPLNNTSSCNC
SPAC6F6.15	Rab GTPase Ypt5	-RPAAQPSGSCSC
SPBC405.04	GTPase Ypt7	-LDMESQKTSCYC
SPAC17A2.10c	*S. pombe* specific protein	-SFRKIASLPCVC
SPBC1703.10	Rab GTPase Ypt1	-GTNVSQSSSNCC
SPAC9E9.07c	Rab GTPase Ypt2	-DLGNDRTVKRCC
SPAC18G6.03	Rab GTPase Ypt3	-DLNKKKSSSQCC
SPAC1B3.11c	Rab GTPase Ypt4	-VRLERQTRSYCC
SPBP4H10.14c	*S. pombe* specific protein	-WKQKLLPLKKCC

[a] The 12 C-terminal amino acids from each sequence are shown. CaaX/CXC/CC motifs are marked in red, with prenylatable residues shown underlined. Palmitoyatable cysteine residues and basic amino acids are shown in blue and green, respectively.

Supplementary Table S2: Strains used in this work

***S.pombe* strains**[a]	**Genotype**	**Source/Reference**
MI200	*h^{+} pmk1-HA6H:$ura4^{+}$*	37
MI201	*h^{-} pmk1-HA6H:$ura4^{+}$*	37
AFS001	*h^{+} mam4::hphR pmk1-HA6H:$ura4^{+}$*	This work
AFS002	*h^{-} mam4::hphR pmk1-HA6H:$ura4^{+}$*	This work
AFS030	*h^{90} mam4::hphR*	This work
MI700	*h^{+} rho2:: kanR pmk1-HA6H: $ura4^{+}$*	38
MI701	*h^{-} rho2:: kanR pmk1-HA6H: $ura4^{+}$*	38
AFS003	*$h^{?}$ rho2:: kanR mam4::hphR pmk1-HA6H: $ura4^{+}$*	This work
AFS004	*$h^{?}$ pmk1:: kanR mam4::hphR*	This work
LSM400	*h^{-} rho2-HA-KSSTKCCIIS: $leu1^{+}$ rho2:: kanR pmk1-HA6H: $ura4^{+}$*	21
AFS005	*$h^{?}$ rho2-HA-KSSTKCCIIS: $leu1^{+}$ rho2:: kanR mam4::hphR pmk1-HA6H: $ura4^{+}$*	This work
LSM500	*h^{-} GFP-rho2-HA-KSSTKCCIIS: $leu1^{+}$ rho2:: kanR pmk1-HA6H: $ura4^{+}$*	21
AFS008	*$h^{?}$ GFP-rho2-HA-KSSTKCCIIS: $leu1^{+}$ rho2:: kanR mam4::hphR pmk1-HA6H: $ura4^{+}$*	This work
LSM501	*h^{-} GFP-rho2-HA-KSSTKSCIIS: $leu1^{+}$ rho2:: kanR pmk1-HA6H: $ura4^{+}$*	21
LSM502	*h^{-} GFP-rho2-HA-KSSTKCSIIS: $leu1^{+}$ rho2:: kanR pmk1-HA6H: $ura4^{+}$*	21
LSM504	*h^{-} GFP-rho2-HA-KSSTKCCIIL: $leu1^{+}$ rho2:: kanR pmk1-HA6H: $ura4^{+}$*	21
AFS009	*$h^{?}$ GFP-rho2-HA-KSSTKCCIIL: $leu1^{+}$ rho2:: kanR mam4::hphR pmk1-HA6H: $ura4^{+}$*	This work
AFS010	*h^{-} GFP-rho2-HA-KKKKKCCIIS: $leu1^{+}$ rho2:: kanR pmk1-HA6H: $ura4^{+}$*	This work
AFS011	*$h^{?}$ GFP-rho2-HA-KKKKKCCIIS: $leu1^{+}$ rho2:: kanR mam4::hphR pmk1-HA6H: $ura4^{+}$*	This work
LSM970	*h^{-} GFP-rho2-HA-RitC: $leu1^{+}$ rho2:: kanR pmk1-HA6H: $ura4^{+}$*	21
AFS012	*$h^{?}$ GFP-rho2-HA-RitC: $leu1^{+}$ rho2:: kanR mam4::hphR pmk1-HA6H: $ura4^{+}$*	This work
AFS100	*h^{-} ras1:: kanR*	This work
AFS101	*h^{-} GFP-ras1: $leu1^{+}$ Ras1:: kanR*	This work
AFS102	*$h^{?}$ GFP-ras1: $leu1^{+}$ Ras1:: kanR mam4::hphR*	This work
AFS013	*h^{-} rho1-HA-TKKKKRCILL: $leu1^{+}$ rho1-596::NatMX6*	This work
AFS014	*h^{-} rho1-HA-TKKKKRCILL: $leu1^{+}$ rho1-596::NatMX6 mam4::hphR*	This work
AFS015	*h^{-} GFP-rho1: $leu1^{+}$ pmk1-HA6H: $ura4^{+}$*	This work
AFS016	*$h^{?}$ GFP-rho1: $leu1^{+}$ mam4::hphR pmk1-HA6H: $ura4^{+}$*	This work
AFS025	*h^{-} GFP-rho1: $leu1^{+}$ rho1-596::NatMX6 pmk1-HA6H: $ura4^{+}$*	This work
AFS026	*h^{-} GFP-rho1-TKKKKCCIIS: $leu1^{+}$ pmk1-HA6H: $ura4^{+}$*	This work
AFS023	*h^{-} GFP-rho1-TKKKKCCIIS: $leu1^{+}$ mam4::hphR pmk1-HA6H: $ura4^{+}$*	This work
YSM2447	*h^{90} cdc42-$sfGFP^{SW}$: kanR*	26
AFS2447	*h^{90} cdc42-$sfGFP^{SW}$: kanR mam4::hphR*	This work
LSM840	*h^{+} erf2:: kanR pmk1-HA6H: $ura4^{+}$*	21
AFS017	*h^{+} erf2:: kanR mam4::hphR pmk1-HA6H: $ura4^{+}$*	This work
AFS37	*h^{+} spk1:: $ura4^{+}$*	This work
LS201	*h^{+} rho1-596::NatMX6 pmk1-HA6H:: ura4+*	27
LS202	*h^{+} rho1-596::NatMX6 rho2:: kanMX6 pmk1-HA6H:: ura4+*	27
AFS027	*$h^{?}$ rho1-596::NatMX6 mam4::hphR pmk1-HA6H:: ura4+*	This work
CA5931	*h^{-} CRIB-GFP:ura4+*	34
AFS018	*$h^{?}$ CRIB-GFP:ura4+ mam4::hphR*	This work
AN0179	*h^{-} psk1-13myc:hphR*	40
AFS019	*$h^{?}$ psk1-13myc:hphR mam4::natR*	This work
PPG6521	*h^{-} HA-cdc42L160S:ura4+*	35

AFS023	*h? mam4::hphR HA-cdc42L160S:ura4+*	This work

[a]All strains are *ade6- leu1-32 ura4D-18*. Substituted amino acids within the natural C-terminal motifs of either Rho1 or Rho2 are shown underlined.
